# Sleep Quality and Cognitive Function after Stroke: The Mediating Roles of Depression and Anxiety Symptoms

**DOI:** 10.3390/ijerph20032410

**Published:** 2023-01-29

**Authors:** Shuzhen Niu, Xianliang Liu, Qian Wu, Jiajia Ma, Songqi Wu, Li Zeng, Yan Shi

**Affiliations:** 1 Tenth People’s Hospital, School of Medicine, Tongji University, Shanghai 200072, China; 2 College of Nursing and Midwifery, Charles Darwin University, Brisbane 4000, Australia; 3 Chest Hospital, Shanghai 200030, China; 4 Tongji Hospital, School of Medicine, Tongji University, Shanghai 200065, China

**Keywords:** ischemic stroke, sleep disturbances, sleep disturbances, anxiety, depression

## Abstract

This study examined the association between post-stroke cognitive function and sleep status at 30 days post-stroke and evaluated the role of anxiety and depression as potential mediators of that association. The participants in this study were 530 acute ischemic stroke (IS) patients. Sleep disturbance at 30 days post-stroke was assessed by the Pittsburgh Sleep Quality Index. Basic patient information, cognitive function, depression, and anxiety status were assessed before discharge from the hospital. Stratified linear regression analysis models were fit to examine the associations between post-stroke sleep quality and the influencing factors. A structural equation model was developed to evaluate the role of anxiety and depression as potential mediators of sleep quality and cognitive function. At 30 days post-stroke, 58.7% of IS patients had sleep disturbance. Women and older IS patients were more likely to suffer poorer sleep quality (*p* < 0.05). A stratified linear regression analysis showed that the inclusion of cognitive function variables and indicators of depression and anxiety were statistically significant in predicting improvement in the sleep disturbance of AIS patients. Cognitive function, depression, anxiety, and sleep status were selected to construct a structural equation model. The total effect of cognitive function on sleep status was −0.274, with a direct effect of −0.097 and an indirect effect (through depression) of −0.177. The total effect of anxiety on sleep status was 0.235, with a direct effect of 0.186 and an indirect effect (through depression) of 0.049. IS patients often experience poor sleep quality. Depression in IS patients mediates two pathways: the pathway through which cognitive function affects sleep quality and the pathway through which anxiety affects sleep quality.

## 1. Introduction

Stroke is one of the major causes of death and disability in the world [1]. Ischemic stroke (IS) accounts for approximately 70% of strokes [2], and its prevalence is increasing year by year [3]. Acute ischemic stroke (AIS) refers to the acute blood supply disorder of local brain tissue, which causes the brain tissue to undergo ischemia and hypoxic necrosis, resulting in neurological dysfunction [4]. IS has a significant impact on patients’ health outcomes and quality of life due to its high rates of morbidity, disability, recurrence, and mortality [5].

Poststroke sleep disturbances (PSSDs) are common following IS. Around 70% of AIS patients and 40% of patients in chronic post-stroke stages experience sleep disorders [6,7]. A large and growing body of literature has indicated that sleep-related problems should be considered in clinical management in addition to cognitive and physical impairments [8]. Sleep disturbances are also risk factors for stroke exacerbation and are strongly associated with the onset and progression of stroke. Although there have been significant advances in research on sleep disorders, PSSD is still underestimated and commonly ignored in clinical diagnosis and treatment [9]. Among adults with stroke, those with poor sleep have a 76% greater risk of early death than those with normal sleep [10]. Unrecognized and untreated sleep disorders may influence rehabilitation efforts, lead to poor functional outcomes following stroke, and increase the risk of stroke recurrence [11]. Data from several studies suggest that sleep and sleep loss bidirectionally alter structural plasticity, which can affect the functional output of the brain in terms of alertness and mood [12].

The existing literature on sleep disorders is extensive and focuses on factors affecting sleep such as age [13], body mass index (BMI) [14], depression [15], and anxiety [16]. Mounting evidence points to a potential connection between sleep and cognitive function [17]. A decline in cognitive function is not only reflected in the values assessed by the physician, but patients may also experience the feeling of cognitive decline in their daily lives. However, few studies have assessed whether cognitive function affects sleep in the months following stroke. Furthermore, little research focuses on the role of depression and anxiety status in the effect of cognitive function on sleep. Hence, there is a need for an investigation of the influence of depression and anxiety on the relationship between cognitive function and sleep disorders. In this study, we propose hypothesis 1: cognitive function affects the sleep quality of IS patients. Additionally, we propose hypothesis 2: Anxiety and depression play a role in the relationship between cognitive function and sleep quality.

## 2. Materials and Methods

### 2.1. Subjects

**Figure 1:**
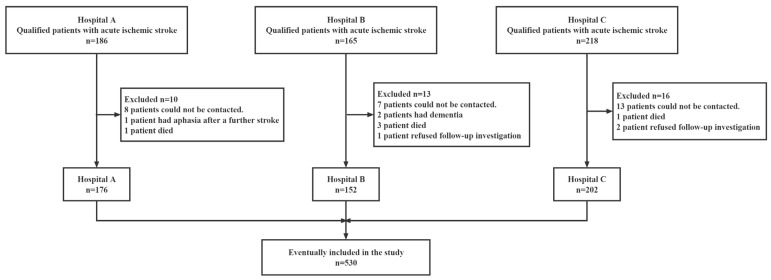
Flowchart of participant enrollment.

A total of 569 adult Chinese patients with AIS were prospectively enrolled from among those listed on inpatient medical care rosters from three tertiary general hospitals in Shanghai from January 2021 to January 2022. Among the 569 patients who had experienced their first IS and had the potential to be included in this study, 39 failed to complete 30 days of data collection, leading to a 6.85% missing rate. The process of patient collection and exclusion is shown in Figure 1. A total of 530 patients were included in the study who completed the baseline data collection and the 30-day post-stroke sleep data collection in full. All patients received a neuroradiological exam (cranial plain CT or MRI (T1/T2/DWI)), and the results were consistent with the “Chinese guidelines for diagnosis and treatment of acute ischemic stroke 2018 [4]”. To avoid the influence of unstable neurological conditions and environmental changes on the results, our investigation was conducted when the patient was conscious and exhibited stable vital signs after routine neurological treatment. A questionnaire on basic patient information, neurological and cognitive function, ADL, and anxiety and depression was administered 5.95 ± 2.12 days after admission to the hospital. All patients returned to their home environment after completing their general hospital treatment. Each patient’s sleep status was followed up by telephone at 30 days after diagnosis, and all questions were answered based on sleep in the past 30 days.

Patients who met any of the following conditions were excluded:

(1) transient ischemic attack (TIA) diagnosed by neurologists;
(2) unable to give appropriate responses to the questions on the questionnaires (altered consciousness, confusion, or aphasia);
(3) diagnosed with dementia of any type or another neurodegenerative or neurological condition.

Ethical approval was obtained from the Ethics Approval Committee of Shanghai Tenth People’s Hospital. All participants provided written informed consent.

### 2.2. Measures

The purpose of this study was to investigate whether cognitive function, anxiety, and depression affect patients’ sleep in the month following stroke and to analyze other factors that may contribute to patients’ sleep disturbance, such as age, activities of daily living (ADL), BMI, and National Institutes of Health Stroke Scale (NIHSS). The Pittsburgh Sleep Quality Index (PSQI) was used to assess the sleep performance of IS patients 30 days after stroke and to identify risk factors for sleep disturbance.

#### 2.2.1. Basic Information Collection and Functional Assessment

The basic information of patients included age, sex, marital status, education level, smoking, alcohol consumption, site of cerebral infarction, height, and weight. In this study, “smoke” was operationally defined as “current smokers” who had smoked within 30 days before the survey. “Drink” was operationally defined as consuming more than 15 g of alcohol per day within 30 days before the survey.

Each patient was assigned an NIHSS score. The NIHSS is used to assess the degree of neurological deficit after a patient has suffered a cerebral infarction. The NIHSS contains judgments on consciousness, speech, movement, sensation, ataxia, eye movement, and visual field. The higher the NIHSS score, the more severe the degree of neurological deficit. Baseline assessment provides a measure of stroke severity, and the effect of treatment can be measured periodically after treatment.

Each patient underwent ADL assessment since ADL competence is one of the most important indicators of the effectiveness of rehabilitation [18]. The Barthel index (BI) is the most commonly used scale in the world to assess ADL competence [19]. A series of studies have shown that BI has high reliability and sensitivity [20]. The BI consists of 10 items: feeding, bed and wheelchair transfer, personal hygiene, toileting, bathing, walking, walking up and down stairs, dressing, bowel control, and urinary control.

#### 2.2.2. Assessment of Sleep Quality

The PSQI was used to measure the subjective sleep quality of AIS patients [21]. The PSQI is a self-reported questionnaire that assesses sleep quality using subjective ratings of 7 components: sleep quality, sleep latency, sleep duration, habitual sleep efficiency, sleep disturbance, use of sleep medication, and daytime dysfunction. The Chinese version of the PSQI was first translated in 1996 by Liu et al. [22]. In the past 20 years, the Chinese version of the PSQI has been widely used in the sleep quality assessment of different populations including college students [23], adolescents [24], nurses [25], older adults [26], and government employees [27]. The results of these studies indicate that the Chinese version of the PSQI is a reliable and valid instrument with internal consistency, test–retest reliability, and high criterion-related validity. The assessment of PSQI was performed by telephone follow-up at 30 days of discharge. Patients fill out the questionnaire based on their sleep status in the past month. The questionnaire has good evidence of validity and reliability [23,24,25]. In this study, patients with PSQI scores ≥ 8 were classified into the poor sleep quality (PSQ) group, and those with scores < 8 were classified into the good sleep quality (GSQ) group.

#### 2.2.3. Assessment of Cognitive Function

The Beijing Revised Montreal Cognitive Assessment Scale (MoCA) [28] was used in this study. This scale consists of 12 items with a total score of 30. The MoCA is divided into eight cognitive subscales: visuospatial/executive function, naming, attention, language, abstraction, memory, and orientation. The assessment of MoCA was performed while conscious and with stable vital signs after routine neurological treatment during hospitalization. The estimated time for the whole procedure is 10–15 min. The maximum score is 30, and lower scores indicate worse cognition. In this study, a score of ≥23 was classified as normal cognitive function and <23 as cognitive impairment [29]. To correct for the bias caused by educational level, a score of 1 was added to the total score for years of education ≤12 years and a score of 2 was added for illiteracy.

#### 2.2.4. Assessment of Post-Stroke Anxiety and Depression

Self-Rating Anxiety Scale (SAS) and Self-Rating Depression Scale (SDS) are psychological scales developed by Zung [30] to measure the severity of anxiety and depression states and how they change during treatment, and they are commonly used psychometric instruments in clinical practice.

### 2.3. Data Analysis

EpiData 3.0 (Odense. Denmark) was used for data entry, and two researchers completed data entry separately to ensure data accuracy. The IBM SPSS statistical software version 22.0 (Armonk, NY, USA) for Windows was used to perform basic descriptive analyses. Descriptive statistics were reported as mean ± standard deviation (SD) for variables with normal distributions and as median (interquartile range, IQR) for variables with skewed distributions. The reliability (internal consistency) was tested using the Cronbach’s alpha coefficient, which indicates the connectedness of items within a scale. Analysis of variance, the chi-squared test, and the rank sum test were used to analyze differences between the GSQ group and the PSQ group for each factor. Stratified linear regression analysis was used to create the regression equations. A *p* value of <0.05 was accepted as statistically significant. The IBM AMOS 23 (Armonk, NY, USA) program was used to analyze the relationships between the constructs involved in the structural model. The bootstrap self-sampling count was set to 5000 for validation. Once the theoretical model was developed, path analysis was performed based on the relationships of the matrix identified via the structural equation analysis.

### 2.4. Patient and Public Involvement

No patients or members of the public were involved in the development of the research question or the design of this study.

## 3. Results

### 3.1. Baseline Characteristics of Patients in the Two Sleep Quality Groups

A total of 530 patients with AIS were included in this study with a 30-day follow-up. To compare baseline characteristics, which are shown in Table 1, the 530 patients were divided into a PSQ group (*n* = 311) and a GSQ group (*n* = 219). A total of 377 men (71.73%) and 153 women (28.87%) were included in the study. The average age was 63.42 (SD = 10.31), and the average BMI was 24.33 (SD = 2.97). Additionally, 145 patients (27.36%) were working before having a stroke. The median values (IQR) of the NIHSS and ADL scores of patients were 3 (3) and 55 (25). In the past year, 217 (40.94%) patients drank alcohol and 204 (38.49%) patients smoked. Hypertension and diabetes were the most common chronic diseases among IS patients, with 325 (61.32%) having hypertension and 241 (45.47%) having diabetes. A comparative analysis of the PSQ group and the GSQ group revealed significant differences in terms of age, sex, drinking, smoking, hypertension, diabetes, SAS scores, SDS scores, and cognitive function.

### 3.2. Assessment of Patients’ Sleep Condition

**Figure 2:**
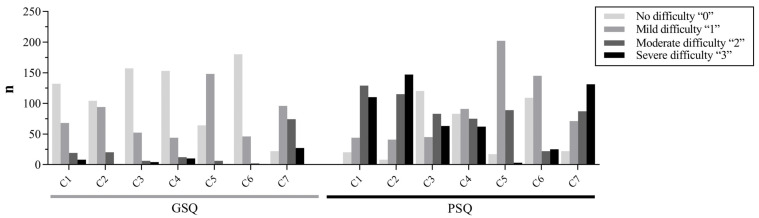
Histogram of the seven components of the PSQI scores for GSQ and PSQ. C1: subjective sleep quality; C2: sleep latency; C3: sleep duration; C4: habitual sleep efficiency; C5: sleep disturbances; C6: use of sleeping medication; C7: daytime dysfunction; GSQ: good sleep quality (*n* = 219); PSQ: poor sleep quality (*n* = 311).

The distribution of the scores on the seven dimensions of PSQI for the PSQ group and GSQ group is shown in Figure 2. The quality of sleep was considered “poor” or “very poor” by 266 (50.19%) patients. Furthermore, 156 (29.43%) patients slept less than 6 h per day, 159 (30%) patients had a sleep efficiency of less than 75%, and daytime dysfunction (sleepiness and low energy) was present in 319 (60.19%) patients.

### 3.3. Association between Sleep Quality and Related Influencing Factors in Is Patients

A stratified linear regression analysis was conducted to examine the association between sleep quality and related influencing factors. The results are shown in Table 2. In this study, the dependent variable, PSQI score, was a continuous variable with a linear relationship between all 12 independent variables. All variables had relatively independent observations, and there was no multicollinearity or significant outliers. Model 1 included PSQI scores and basic characteristics of IS patients. Based on the model 1 variables, neurological deficits (NIHSS) and ADL competence (BI) were added to create model 2. In this study, model 2 differed from model 1 only in the NIHSS and BI scores, suggesting that the inclusion of NIHSS and BI scores in the regression increased the explanatory power of the independent variables for the PSQI variance by 2.3%. Based on model 2, cognitive function (MOCA) variables were added to create model 3. Model 3 showed a 4.4% increase in the explanatory power of independent variables for the variance in PSQI. Based on model 3, depression (SDS) and anxiety (SAS) variables were added to create model 4. Model 3 showed a 21% increase in the explanatory power of independent variables for the variance in PSQI.

### 3.4. The Mediation Role of Depression (SDS) between MoCA, SAS, and PSQI

**Figure 3:**
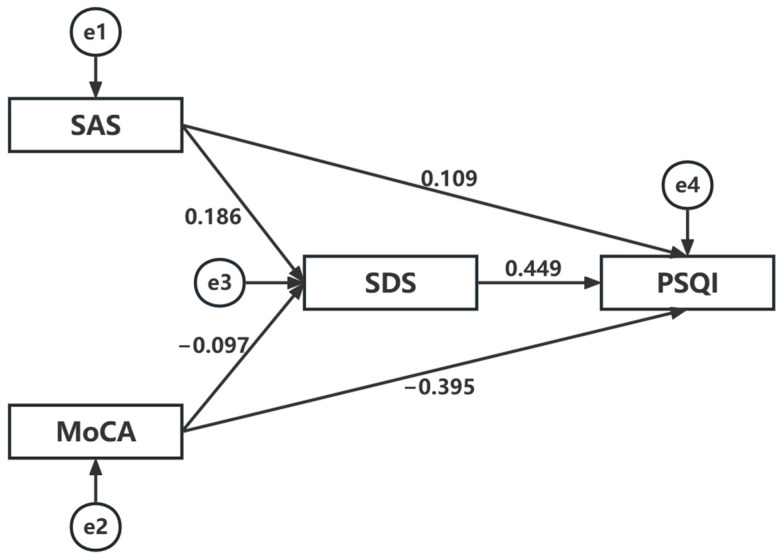
Mediating effects of depression (SDS score) on the relationship between cognitive function (MoCA score) and sleep quality (PSQI score) and on the relationship between anxiety (SAS score) and sleep quality (PSQI score).

With the above statistical results, structural equation modeling was used to construct a pathway analysis of the interaction between depression (SDS), cognitive function (MoCA), anxiety (SAS), and sleep quality (PSQI). The initial model was tested using the maximum likelihood method, and the model fit parameters were as follows: X^2^/df = 1.242, P = 0.265, NFI = 0.996, RFI = 0.973, IFI = 0.999, CFI = 0.999, GFI = 0.999, TLI = 0.995, AGFI = 0.988, and RMSEA = 0.021, all of which met statistical criteria. There are two pathways through which cognitive function affects sleep quality. One is that cognitive function directly affects sleep quality, and the other is that cognitive function indirectly affects sleep quality by affecting depression. The standardized total effect of MoCA on PSQI was −0.274, with a standardized direct effect (PSQI←MoCA) of −0.097 and a standardized indirect effect (PSQI←SDS←MoCA) of −0.177. There are two pathways through which anxiety affects sleep quality. One is that anxiety directly affects sleep quality, and the other is that anxiety indirectly affects sleep quality by affecting depression. The standardized total effect of SAS on PSQI was 0.235, with a standardized direct effect (PSQI←SAS) of 0.186 and a standardized indirect effect (PSQI←SDS←SAS) of 0.049. The final model is presented in Figure 3. The regression weights for each variable are shown in Table 3. The above model was further tested using the bootstrap bias-corrected self-help method. The results are shown in Table 4.

## 4. Discussion

Given the significant role of PSSD in the prognosis of IS, this study aimed to determine whether sleep quality is affected by variables, such as age, BMI, sex, ADL competence, cognitive function, depression, and anxiety. This study focused on analyzing the association between PSSD and cognitive function after stroke and determining whether depression and anxiety mediated this association. The results of the regression analysis and structural equation model revealed that depression (SDS scores) and cognitive function (MoCA scores) best explained sleep quality.

The results of this study indicate that IS patients often experience poor sleep quality. As was mentioned in the literature review [31], PSSD is frequently reported after stroke, occurring in 21–77% of stroke patients. Sleep disturbances show a dynamic prevalence across different stroke phases. The literature points out that the prevalence rates of insomnia in the acute, subacute, and chronic phases were 40.7%, 42.6%, and 35.9% (95% CIs, 31.8–50.3, 31.7–54.1, and 28.6–44.0), respectively, when evaluating self-reported insomnia symptoms by means of questionnaires [32]. In this study, the prevalence of poor sleep quality in patients one month after stroke was 58.68%. The results obtained for the PSQI score suggest that IS patients from the PSQ group were more likely to experience sleep disturbance and exhibit higher sleep latency, sleep duration, more use of sleeping medication, and daytime dysfunction when compared with the GSQ group on average.

Women and older IS patients were more likely to develop sleep disorders in our study. Since the age of stroke onset coincides with the age of perimenopause in women, fluctuating or decreasing estrogen levels in women with IS can indirectly affect the sleep–wake cycle, altering sleep patterns and leading to sleep disorders [33,34]. Some studies have found that sleep disorders are common in patients with a high degree of neurological deficits [35]. Kim et al. [9] found a significant association between functional status (mRS) and sleep quality, insomnia, and excessive daytime sleepiness. However, our study did not find differences in NIHSS scores or BI scores between the GSQ and PSQ groups. The discrepancy between our results and those of Kim et al. may be related to the selection of assessment methods and evaluation indicators.

An important finding of our study was that IS patients from the PSQ group also had lower cognitive function scores compared with the GSQ group. The pathway of influence from cognitive function to sleep quality involved depression. Our findings are consistent with prospective data from 2474 older white women followed for 15 years showing that cognitive decline is associated with sleep disturbance in nondemented community-dwelling older women [36]. When patients with IS have poor cognitive function, they may experience a decline in memory, language function, or attention. This cognitive decline can cause a great deal of psychological stress. If a patient cannot accept cognitive decline, they may become depressed. Strong empirical evidence suggests that sleep deprivation can have serious consequences on cognitive function [37]. Neurocognitive impairments caused by poor sleep are linked to the reduced functionality of the prefrontal cortex [38]. Studies suggest that shared mechanisms underlie circadian rhythmicity and long-term memory formation [39]. The Sleep Study Group of the Italian Dementia Research Association (SINDem) conducted a multicenter study on the prevalence of sleep disturbances in mild cognitive impairment and dementia disorder patients. The study indicated that two or more sleep disturbances almost always occur in association in the same patient [40]. This suggests that there is a two-way relationship between cognitive function and sleep quality.

One of the more significant findings to emerge from our study was that depression in IS patients mediated two pathways: the pathway through which cognitive function affects sleep quality and the pathway through which anxiety affects sleep quality. This also confirms that depression is an important factor associated with sleep disorders in individuals with stroke [41]. Saper et al. [42] noted that an important but often overlooked factor affecting sleep is mood, including stress and depression, which can be the origin and pathophysiology of the predisposition to insomnia. In addition, Drake et al. [43] suggested that other personality traits related to emotion were also associated with insomnia, including neuroticism, perfectionism, and sensitivity to anxiety symptoms. In this study, depression and anxiety in IS patients significantly affected sleep quality and explained 21% of the occurrence of sleep disturbances after excluding all other factors from the study. In the final structural equation, depression played a mediating role in the effect of anxiety on sleep disturbance. All patients included in this study had experienced their first stroke. A stroke is a devastating blow to the patient and may drastically change the patient’s life and psychological status. In the first month following a stroke in particular, patients suffer from various complications caused by the stroke, including physical dysfunction. Depression and anxiety are very common in stroke patients. One study found that 31% of stroke survivors reported depression within 5 years after having a stroke [44]. Depression is associated with sleep quality at both the acute [41] and chronic [45,46] post- stroke stages. For stroke survivors, depression is significantly associated with poorer functional status and rehabilitation outcomes, which are detrimental to patient health outcomes and quality of life [47,48]. It has been confirmed that risk genes seem to have a preference for brain circuitries involved in emotion regulation. The brain tissues and cell types expressing sets of insomnia risk genes are not primarily part of the known circuitry regulating sleep but are rather part of circuitries involved in emotion regulation [49].

The latest evidence suggests that if sleep disorders are accurately diagnosed in the early post-stroke stages, appropriate treatments can be administered early on, leading to the recovery of functional impairment and the improvement of social participation. Many studies emphasize and advocate the importance of collaboration between sleep specialists, neurologists, clinicians, caregivers, and rehabilitation specialists to expand our knowledge of this field and bring a restful night’s sleep to IS patients.

There are several limitations to this study. The subjects were not tested with Polysomnography in this study. There could be unidentified sleep disorders that cannot be screened for via questionnaires. An issue that was not addressed in this study was that damaged brain areas following a stroke handle different features of sleep quality. We did not investigate the association between sleep disorders and the anatomical locations of stroke lesions. Finally, because the information was self-reported, the data were prone to information bias.

## 5. Conclusions

Sleep disorders are largely underestimated and do not receive sufficient attention in clinical practice. The prevalence of new-onset sleep disorders after stroke was 58.68% in this study. Sleep disturbances are significantly affected by cognitive decline and may be associated with significant psychological distress and depression. Early identification should be conducted and targeted measures should be taken to reduce depression levels in IS patients presenting with cognitive decline. As Prof. J. McKinley wrote, “when we try to encourage a good night’s sleep for our patients, perhaps we are doing them more good than we thought” [50].

## Figures and Tables

**Table 1:** Sociodemographic characteristics of the participants (*n* = 530).

Variables	Good Sleep Quality (*n* = 219)	Poor Sleep Quality (*n* = 311)	Statistics	*p*
Age	62.15 ± 10.63	64.31 ± 9.99	5.706	0.017
BMI	24.06 ± 2.96	24.52 ± 2.96	3.132	0.077
Sex, *n* (%)				
Male	172 (78.5)	205 (65.9)	9.971	0.002
Female	47 (21.5)	106 (34.1)		
Marriage, *n* (%)				
Married	200 (91.3)	279 (89.7)	0.385	0.535
Unmarried/divorced/Widowed	19 (8.7)	32 (10.3)		
Education years, *n* (%)				
Under primary school (≤6)	17 (7.8)	41 (13.2)	5.368	0.147
Primary school (7–9)	86 (39.3)	130 (41.8)		
High school (10–12)	60 (27.4)	74 (23.8)		
University and above (≥13)	56 (25.6)	66 (21.2)		
Working status, *n* (%)				
Employed	62 (28.3)	83 (26.7)	0.170	0.680
Retired	157 (71.7)	228 (73.3)		
Medical insurance, *n* (%)				
Yes	191 (87.2)	276 (88.7)	0.288	0.592
No	28 (12.8)	35 (11.3)		
Vascular risk factors, *n* (%)				
Drink	77 (35.2)	140 (45.0)	5.163	0.023
Smoke	73 (33.3)	131 (42.1)	4.193	0.041
Hypertension	120 (54.8)	205 (65.9)	6.702	0.010
Diabetes	87 (39.7)	154 (49.5)	4.969	0.026
Coronary heart disease	26 (11.9)	38 (12.2)	0.015	0.904
Atrial fibrillation	12 (5.5)	15 (4.8)	0.114	0.735
Hyperlipidemia	45 (20.5)	66 (21.2)	0.035	0.851
Hyperuricemia	13 (5.9)	21 (6.8)	0.143	0.706
Thyroid disease	17 (7.8)	26 (8.4)	0.062	0.804
TIA history	14 (6.4)	28 (9.0)	1.200	0.273
NIHSS (M, IQR)	3 (3)	3 (3)	−0.313	0.754
BI (M, IQR)	55 (30)	55 (25)	−0.479	0.632
SAS	32.50 ± 6.25	35.26 ± 8.34	17.208	***
SDS	36.14 ± 7.53	43.29 ± 11.51	64.778	***
MoCA (M, IQR)	22 (5)	21 (8)	−4.901	***

*** Correlation is significant at the 0.001 level (two-tailed).

**Table 2:** Association between sleep quality and related influencing factors in IS patients.

Variables	Model 1		Model 2		Model 3		Model 4	
	*β*	*SE*	*β*	*SE*	*β*	*SE*	*β*	*SE*
Constant	6.873 ***	1.271	5.615 ***	1.457	9.300 ***	1.569	6.519 ***	1.647
Age	0.055 ***	0.016	0.054 ***	0.016	0.036 *	0.016	0.015	0.014
Sex	−1.335 ***	0.361	−1.321 ***	0.258	−1.249 ***	0.350	−0.775 *	0.308
Marriage	−0.604	0.551	−0.629	0.545	−0.381	0.535	−0.507	0.468
EDU	−0.065	0.171	−0.037	0.169	0.321	0.174	0.113	0.153
BMI	0.125	0.176	0.136	0.174	0.234	0.174	0.121	0.154
Work	−0.170	0.119	−0.186	0.119	0.125	0.146	0.027	0.128
Insurance	0.067	0.188	0.141	0.187	0.159	0.199	0.128	0.245
NIHSS			0.258	0.071	0.219 **	0.070	0.101	0.062
BI			−0.249 *	0.103	−0.260 *	0.102	−0.120	0.106
MoCA					−0.204 ***	0.040	−0.111 **	0.037
SAS							0.087 ***	0.018
SDS							0.167 ***	0.015
R^2^	0.056		0.079		0.123		0.333	
Adj R^2^	0.049		0.069		0.111		0.322	
ΔR^2^			0.023		0.044		0.210	
ΔF	7.765 ***		6.710 ***		25.939 ***		81.969 ***	

*** Correlation is significant at the 0.001 level (two-tailed). ** Correlation is significant at the 0.01 level (two-tailed). * Correlation is significant at the 0.05 level (two-tailed).

**Table 3:** Direct effects of variables.

Items	Estimate	*S.E.*	*t*	*p*	Standardized Estimate
SDS←MoCA	0.918	0.092	−9.973	***	−0.395
SDS←SAS	0.152	0.055	2.758	0.006	−0.109
PSQI←MoCA	−0.080	0.033	−2.438	0.015	−0.097
PSQI←SDS	0.161	0.014	11.241	***	0.449
PSQI←SAS	0.093	0.018	5.066	***	0.186

*** Correlation is significant at the 0.001 level (two-tailed).

**Table 4:** Total, direct, and mediated effects on PSQI.

Items	*β*	*S.E.*	*95%CI*	*p*	Variance (%)
Total effect					-
PSQI←MoCA	−0.274	0.036	(−0.334, −0.215)	***	-
PSQI←SAS	0.235	0.040	(0.165, 0.297)	***	-
Direct effect					-
PSQI←MoCA	−0.097	0.038	(−0.160, −0.034)	***	35.4 ^a^
PSQI←SAS	0.186	0.037	(0.120, 0.245)	0.015	79.1 ^b^
Indirect effect					-
PSQI←SDS←MoCA	−0.177	0.023	(−0.214, −0.139)	***	64.6 ^a^
PSQI←SDS←SAS	0.049	0.017	(0.021, 0.079)	0.004	20.9 ^b^

^a^: Percentage of standardized total effects of PSQI←MoCA. ^b^: Percentage of standardized total effects of PSQI←SAS. *** Correlation is significant at the 0.001 level (two-tailed).

## Data Availability

Data are available upon reasonable request. Data are available upon request from the corresponding author.
